# HPLC-DAD Analysis, Antileishmanial, Antiproliferative, and Antibacterial Activities of *Lacistema pubescens*: An Amazonian Medicinal Plant

**DOI:** 10.1155/2014/545038

**Published:** 2014-08-07

**Authors:** Josiane Mello da Silva, Luciana Maria Ribeiro Antinarelli, Nícolas de Castro Campos Pinto, Elaine Soares Coimbra, Elaine Maria de Souza-Fagundes, Antônia Ribeiro, Elita Scio

**Affiliations:** ^1^Bioactive Natural Products Laboratory, Department of Biochemistry, Biological Sciences Institute, Federal University of Juiz de Fora, José Lourenço Kelmer, s/n, São Pedro, 36036-900 Juiz de Fora, MG, Brazil; ^2^Department of Parasitology, Microbiology and Immunology, Biological Sciences Institute, Federal University of Juiz de Fora, Juiz de Fora, MG, Brazil; ^3^Department of Physiology and Biophysics, Biological Sciences Institute, Federal University of Minas Gerais, Avenida Antônio Carlos, 6627, Pampulha, 31270-901 Belo Horizonte, MG, Brazil

## Abstract

Species of the genus *Lacistema* are traditionally used by Brazilian and Peruvian indigenous communities. The present study investigated the *in vitro* antileishmanial activity against several *Leishmania* species, cytotoxicity in murine peritoneal macrophages, antiproliferative activity against HL60 and Jurkat cells, and antibacterial activities against seven bacteria strains of the aerial parts of the methanolic crude extract and fractions of *Lacistema pubescens*. In addition, their chemical profile was also evaluated. Hexane fraction showed the most significant IC_50_ values against all promastigotes of *Leishmania* species tested, except for *L. chagasi* (IC_50_ = 4.2 *µ*g/mL for *L. major* and IC_50_ = 3.5 *µ*g/mL for *L. amazonensis*). This fraction also exhibited a strong activity against amastigotes of *L. amazonensis* (IC_50_ = 6.9 *µ*g/mL). The antiproliferative activity was also observed for methanolic extract and hexane fraction with IC_50_ = 47.2 *µ*g/mL and IC_50_ = 39.7 *µ*g/mL for HL60, respectively. Regarding the antimicrobial activity, the overall antibacterial activity was not very significative. Phytol and sitosterol were identified in the methanolic extract. Additionally, previous studies also revealed the presence of those compounds in the hexane fraction. Among other compounds, phytol and sitosterol were probably involved in the antileishmanial and cytotoxicity activities observed in this study.

## 1. Introduction

The family Lacistemataceae is represented by the genera* Lacistema* and* Lozania* with about 11 and 4 species, respectively [1]. Some species of* Lacistema* are traditionally used by Brazilian indigenous from Amazon against rheumatism, vomiting, dysentery, body aches, and fever [2–4]. A list of the Peruvian Amazonian plants used for medicinal purposes also mentioned* Lacistema* sp. to combat rheumatism and as antipyretic [4]. In addition, some studies had shown the potential pharmacological properties of some* Lacistema *species such as antiplasmodial [4], antimutagenic [5], antifungal [6], and antiviral [7].

Originally from Brazil,* Lacistema pubescens* Mart. is widely distributed in other countries such as Bolivia, Guyana, and Venezuela. In Brazil, it is known as “espeto vermelho,” “canela vermelha” [8, 9] “sabãozinho” [10], and “cafezinho” [11]. Recently, it was evidenced that the leaves of* L. pubescens* present a potential antioxidant capacity, possibly correlated to phenolic compounds detected in this species [12]. Also, the antinociceptive and anti-inflammatory activity of the hexane fraction of* L. pubescens *leaves were reported [13] confirming the popular use of the genus by indigenous communities. This study also resulted in the identification of fatty acids such as palmitic acid and stearic and linoleic acid in this fraction. Tocopherol, the diterpene phytol, and the sterol sitosterol were also identified.

To the best of our knowledge, apart from those preliminary reports on the chemical composition and pharmacological activities of* L. pubescens* [12, 13], no other reports on the phytochemical or bioactivity of this species are available to date. So, the present study was conducted to evaluate the* in vitro* antibacterial, antiproliferative, and antileishmanial properties and to identify some compounds of the methanolic extract of* L. pubescens.*

## 2. Materials and Methods

*Plant Material*.* Lacistema pubescens* Mart. leaves were collected in Juiz de Fora, State of Minas Gerais, Brazil, in December 2011 and identified by Dra. Fátima Regina Gonçalves Salimena from the Department of Botany of the Institute of Biological Sciences (Federal University of Juiz de Fora). A voucher specimen (CESJ 49751) has been deposited at the Leopoldo Krieger Herbarium of the Federal University of Juiz de Fora.

*Preparation of the Methanolic Extract and Fractions*. The dried leaves (375 g) were powdered and macerated with methanol (5 × 300 mL) for five days at room temperature. The methanolic extract (57 g), after removal of solvent, was dissolved in MeOH-H_2_O (8 : 2) and partitioned with hexane and ethyl acetate to obtain hexane (16 g), ethyl acetate (7 g), and the remaining hydromethanolic fractions (5 g). The extract and fractions were then concentrated using a rotary evaporator under reduced pressure and kept in tightly stoppered bottle under refrigeration until being used for the biological testing.

*High Pressure Liquid Chromatography (HPLC) Analysis.* HPLC analysis with a DAD detector and an automatic injector (Agilent Technologies 1200 Series, USA) was performed for methanolic extract, ethyl acetate, and hydromethanolic fractions. The column employed was a Zorbax SB-18; 250 × 4.6 mm, 5 *μ*m particle size. The mobile phase applied was 0–30 min, HPLC-grade methanol-acetonitrile (70 : 30 v/v). The sample volume was 20 *μ*L (1 mg/mL), the flow rate was 1 mL/min, and the temperature was maintained at 25°C during the analysis. Detection was performed at 210 nm. Phytol and sitosterol standards (Sigma-Aldrich, USA) were used in this experiment as markers under the same conditions used for the samples.

*In Vitro Antileishmanial Activity Evaluation*.* Leishmania major *(MRHO/SU/59/P),* L. braziliensis *(MHOM/BR/M2903),* L. chagasi* (MHOM/BR/PP75), and* L. amazonensis *(IFLA/BR/67/PH8) promastigotes were used for* in vitro* screening.

*Antipromastigote Assays.* The antileishmanial activity was determined by colorimetric 3-(4,5-dimethylthiazol-2-yl)-2,5-diphenyltetrazolium bromide (MTT, Sigma-Aldrich, USA) method [14]. Briefly, promastigotes of* L. amazonensis* were cultured in the Warren's medium (brain heart infusion plush hemin and folic acid), promastigotes of* L. braziliensis* and* L. major* were maintained in BHI medium, and promastigotes of* L. chagasi *were maintained in medium 199, both supplemented with 10% fetal bovine serum at 24°C. The screening was performed in 96-well microplates maintained at 24°C. The samples tested were added at different concentrations (100 to 6.25 *μ*g/mL) after dilution in water or DMSO (dimethylsulfoxide), and the highest used concentration of DMSO was 0.8% (v/v), which is not toxic to the parasites. Then, a parasite suspension from a logarithmic phase culture was suspended to yield 2 million parasites/well* (L. amazonensis)* or 3 million parasites/well (*L. chagasi, L. major, and L. braziliensis*) after Neubauer chamber counting. Controls with DMSO and without plant samples were performed. All samples were tested in triplicate, in three independent experiments. The viability of promastigotes was assayed after a three-day incubation period with addition of MTT. The reaction was stopped with HCl in isopropyl alcohol and the optical densities were evaluated in a spectrophotometer at 570 nm. The results were expressed as the concentrations inhibiting parasite growth by 50% (IC_50_) calculated using a nonlinear regression curve, by using GraFit Version 5 software (Erithacus Software, Horley, UK). Amphotericin B (Cristalia, São Paulo, Brazil) was used as the standard drug (1.000 to 0.008 *μ*g/mL).

*Antiamastigote Assays.* Macrophages were obtained from the peritoneal cavity of BALB/c mice previously inoculated with 3% thioglycollate medium (Sigma-Aldrich, USA). Briefly, peritoneal macrophages were plated at 2 × 10^6^ cells/mL on coverslips (13 mm diameter) previously arranged in a 24-well plate in RPMI 1640 medium supplemented with 10% inactivated FBS, and allowed to adhere for 24 h at 33°C in 5% CO_2_. Adherent macrophages were infected with* L. amazonensis *(IFLA/BR/67/PH8) promastigotes in the stationary growth phase using a 1 : 10 ratio at 33°C for 4 h. Noninternalized promastigotes were eliminated and solutions of test compounds were added (25.0 to 1.6 *μ*g/mL) and maintained at 33°C 5% CO_2_ for 72 h. Slides were fixed and stained with Giemsa for parasite counting (optical microscopy, 1000x magnification). The parasite burden was evaluated was evaluated comparing the number of intracellular amastigotes in treated and untreated cultures (number of amastigotes in treated group/number of amastigotes in untreated group × 100). A minimum of 200 cells were counted (infected and uninfected macrophages). Miltefosine (Cayman Chemical Company, Michigan, USA) was used as the reference drug (1.000 to 0.039 *μ*g/mL). All procedures were performed in agreement with the Ethical Principles in Animal Research and according to protocols approved by the Ethical Committee of for Animal Research.

For statistical analysis, the 50% inhibitory concentration, that is, the minimum compound concentration that caused 50% reduction in survival of the amastigote and promastigote forms of* Leishmania* (IC_50_), was carried out and the 95% confidence intervals were included, calculated by Litchtfiet and Wilcoxon method using the Probit. The graphs were plotted by the program GraphPad Prism 4 (GraphPad software, USA). The data were analyzed statistically using analysis of variance (ANOVA) followed by Dunnett post-test to compare all groups to the control group. Differences were regarded as significant when *P* < 0.0001 (^∗∗∗^).

*Cytotoxicity against Mammalian Cells. *Mouse peritoneal macrophages were plated at 2 × 10^6^ macrophages/mL in 96-well culture plates and incubated for 72 h at 37°C in 5% CO_2_ in RPMI 1640 medium containing 10% fetal bovine serum and different concentrations of the test samples (150.0 to 9.4 *μ*g/mL) in 0.5% DMSO. The viability of the macrophages was determined with the MTT assay and was confirmed by comparing the morphology of the control group via light microscopy. CC_50_ values (50% cytotoxicity concentration) were obtained by using GraFit Version 5 software. The cytotoxicity for macrophages and for amastigotes of* L. amazonensis* was compared using the selectivity index (SI), which was determined as the ratio between CC_50_ for macrophages and IC_50_ for parasites.

*Cell Lines. *HL60 cells (human promyelocytic leukemia HL-60 cells) and human immortalized lines of T lymphocyte (Jurkat cells) were a kind gift from Dr. Gustavo Amarante-Mendes (São Paulo University, Brazil). All lineages were cultivated in the logarithmic phase of growth in RPMI 1640 medium (Sigma-Aldrich, USA) supplemented with 100 U/mL penicillin and 100 *μ*g/mL streptomycin enriched with 2 mM of L-glutamine and 10% of fetal bovine serum (Gibco). All cultures were maintained at 37°C in a humidified incubator with 5% CO_2_.

*Cytotoxic Effect against Human Tumor Cell Lines. *The leukemia cell lines were cultured in 96-well plate at densities of 50,000 cells/well, in a final volume of 200 *μ*L/well. The plates were preincubated in a 5% CO_2_/95% air-humidified atmosphere at 37°C for 24 h to allow adaptation of cells prior to the addition of the samples. The test samples were dissolved in DMSO and the half maximal inhibitory concentration (IC_50_) was determined over a range of concentrations of a freshly prepared solution of the samples (100 to 6.25 *μ*g/mL). All cell cultures were incubated in a 5% CO_2_/95% air-humidified atmosphere at 37°C for 48 h. Cell viability was estimated by MTT assay [14]. The optical densities were measured with a spectrophotometer at 590 nm. Control groups included treatment with 0.1% DMSO (negative control) and etoposide 14 *μ*M (positive control). All samples were tested in triplicate, in three independent experiments.

*Antibacterial Assay. *The samples were evaluated against a panel of bacteria strains, including* Staphylococcus aureus* (ATCC 6538),* Pseudomonas aeruginosa* (ATCC 15442),* Shigella dysenteriae* (ATCC 13313),* Salmonella typhimurium* (ATCC 13311),* Escherichia* coli (ATCC 10536),* Enterococcus faecalis* (ATCC 51299), and* Enterobacter cloacae* (ATCC 10699).

*Serial Dilution Assay for Determination of the Minimal Inhibitory Concentration (MIC). *The minimal inhibitory concentration (MIC) of the extract and its fractions was determined by using the broth microdilution technique [15]. MIC values were determined in Mueller Hinton broth (MHB). Bacteria were cultured overnight at 37°C for 24 h in BHI. Sample stock solutions were diluted from 1000 to 7.8 *μ*g/mL (final volume = 80 *μ*L) and a final DMSO concentration ≤1%. Then, MHB (100 *μ*L) was added into microplates. Finally, 20 *μ*L of 10^8^ CFU/mL (according to McFarland turbidity standards) of standardized bacterial suspensions was inoculated into microplates and the test was performed at a volume of 200 *μ*L. Plates were incubated at 37°C for 24 h. The same tests were performed simultaneously for growth control (MHB + bacteria) and sterility control (MHB + extract/fractions) and as positive control, chloramphenicol at concentrations from 100 to 0.78 *μ*g/mL. MIC was calculated as the lowest concentration which shows complete inhibition of the strain tested.

## 3. Results and Discussion

**Figure 1:**
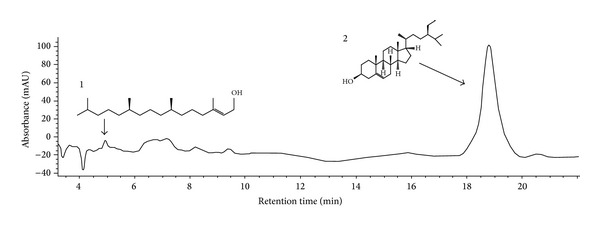
Chromatogram of methanolic extract of* L. pubescens *leaves. Peaks identified: 1, phytol (TR = 4.8 min), and 2, sitosterol (TR = 18.5 min). HPLC conditions: Zorbax SB-18 (250 mm × 4.6 mm i.d.; 5 *μ*m); methanol-acetonitrile (70 : 30 v/v); 1.0 mL/min; injection volume, 20 *μ*L; UV detection at 210 nm.

The HPLC chromatogram profile of the methanolic extract of* L. pubescens* was performed and two compounds were identified as phytol and sitosterol (Figure 1). Those compounds were not detected in ethyl acetate and hydromethanolic fractions (data not shown). Additionally, our previous studies also revealed the presence of those compounds in the hexane fraction [13].

The methanolic extract of* L. pubescens *leaves and its fractions were tested against promastigote forms of different* Leishmania* species which are responsible for various clinical manifestations, ranging from simple cutaneous form to severe visceral form. This is a widespread disease, affecting 12 million people around the world with about 1-2 million estimated new cases occurring every year [16]. The current treatment for all forms of clinical manifestation of leishmaniasis is based on pentavalent antimonials, amphotericin B, and Pentamidine. However, they are toxic, expensive, and difficult to administer and their use induces parasite resistance [17, 18].

For all bioassay, the IC_50_ values below 100 *μ*g/mL were considered significant [19]. As demonstrated in Table 1, the methanolic extract and hexane fraction were potent against* L. amazonensis*, with IC_50_ = 3.9 and 3.5 *μ*g/mL, respectively. Hexane fraction also presented strong activity for* L. major* (IC_50_ = 4.2 *μ*g/mL), while methanolic extract was completely inactive. For* L. braziliensis*, those samples showed IC_50_ = 17 and 45.6 *μ*g/mL, respectively. Ethyl acetate and hydromethanolic fractions presented no antileishmanial activity. Neither the extract nor the fractions were active against* L. chagasi* promastigotes at the maximum concentration tested. Variations in sensitivity to several pure compounds and extracts against promastigotes of different* Leishmania* species have also been reported in previous studies [20–24]. Biochemical and molecular differences between* Leishmania* species including the pattern of glycosylation, enzymes as nucleotidases, and phosphomonoesterases, as well as the major lipophosphoglycan (LPG) present in surface of* Leishmania* sp., have been reported [25–27]. Comparison between the whole-genome expression patterns of* L. major* and* L. infantum *also showed some variations in genes involved in metabolism, cellular organization and biogenesis, transport and genes encoding unknown function [28]. Thus the variation in intrinsic sensitivity to several extracts is not surprising.

**Figure 2:**
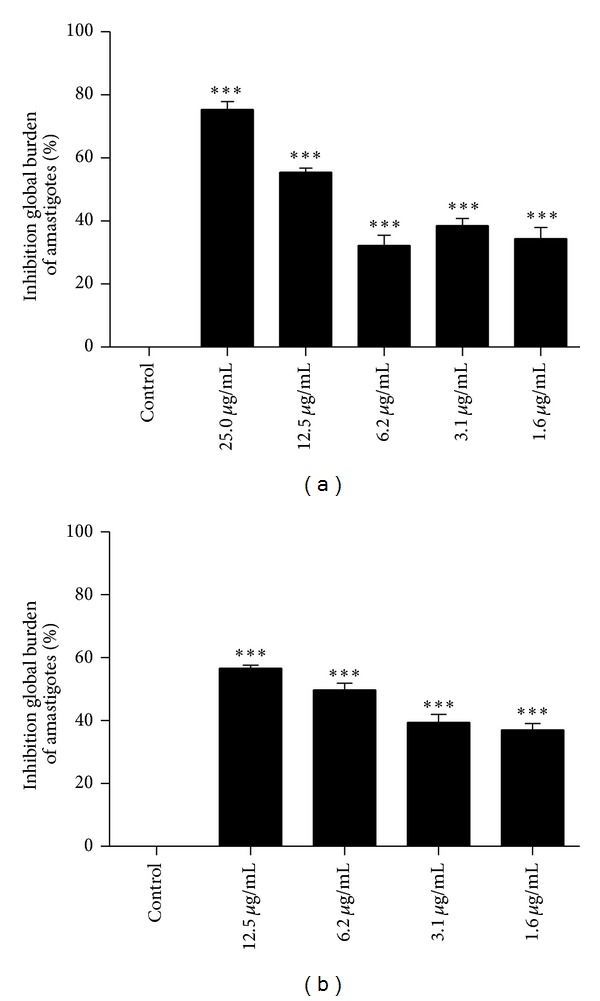
Effect of methanolic extracts (a) and hexane fraction (b) on intracellular amastigotes. Peritoneal macrophages previously infected with* L. amazonensis* promastigotes in the stationary growth phase were exposed to the samples for 72 h. The parasite burden was evaluated comparing the number of intracellular amastigotes in treated and untreated cultures (control). All results were significant (***P < 0.0001).

Promastigote forms of* Leishmania* were used as preliminary screening for antileishmanial activity. Subsequently, assays using amastigote forms were also performed. Intracellular amastigotes are considered the gold standard for antileishmanial* in vitro* evaluation, as this parasite forms are found in mammalian host, including the man, and are responsible for all clinical manifestations of leishmaniasis [29].* Leishmania amazonensis*, the most sensitive* Leishmania* species in the antipromastigote tests (Table 1), was chosen for the antiamastigote assays. In this assay, the macrophages were efficiently infected (controls = 50% of infected macrophages, with 4.3 parasites/infected cells). In general, it was observed that the methanolic extract and hexane fraction of* L. pubescens *showed dose-dependent effect in the reduction of the number of intracellular parasites (Figure 2). Furthermore, as can be seen in Figure 2, hexane fraction showed a higher percentage of inhibition of global burden of amastigotes with 56.6, 49.7, 38.4, and 37.1% (12.5, 6.2, 3.1, and 1.6 *μ*g/mL, resp.). Methanolic extract also exhibited expressive activity with 75.4, 55.5, 32.3, 38.5, and 34.4% of inhibition of global burden (25.0, 12.5, 6.2, 3.1, and 1.6 *μ*g/mL, resp.). Furthermore, IC_50_ values for the hexane fraction and methanolic extract against* L. amazonensis* amastigotes was very low (6.9 and 8.1 *μ*g/mL, resp.) and highlighted the activity of those samples against intracellular form of the parasite (Table 2). Miltefosine, the reference drug, exhibited IC_50_ value of 3.9 *μ*g/mL.

Macrophages are the main host cell for* Leishmania *protozoa, so it is important to know the toxicity of the methanolic extract and hexane fraction of* L. pubescens* on these cells as well as their selectivity on the intracellular parasites. Compounds with selectivity index (SI) ≥ 1.0 were considered more destructive to amastigotes of* L. amazonensis* than to the host cells [30]. Regarding to the selectivity of the samples, it is important to be mentioned that, despite the cytotoxic effect on macrophages, methanolic extract and hexane fraction were 4.1 and 3.6 times, respectively, more destructive to amastigote than to the host cells (Table 2).

Sitosterol was identified as one of the chemical constituents detected in methanolic extract of* L. pubescens* leaves (Figure 1) and in the hexane fraction [13]. This compound had been already shown to have antileishmanial activity by inhibiting in 70% the promastigote forms of* L. amazonensis* growth at 100 *μ*g/mL [31]. So, there is a possibility that sitosterol may be partially responsible for the antileishmanial activity of* L. pubescens*. However, the leishmanicidal activity against promastigotes and amastigotes of* L. amazonensis* observed in this work may be also attributed to other compounds present in active samples.

Natural extracts have been previously reported as potential sources of antiproliferative compounds [32–34]. The antiproliferative activity of methanolic extract and fractions of* L. pubescens* against HL60 e Jurkat human leukemia cells was evaluated and the results are shown in Table 1. Methanolic extract and the hexane fraction showed a moderate activity against HL60 cells while Jurkat cells were resistant to all samples tested.

Compounds like phytol and sitosterol, found in the methanolic extract and previously detected in the hexane fraction [13] may be, at least in part, responsible for the antiproliferative activity against HL60 cell line. Studies have demonstrated that sitosterol showed a highest antiproliferative activity against human tumor cells [35]. Results suggested that sitosterol induces endoreduplication by promoting spindle microtubule dynamics through the Bcl-2 and PI3 K/Akt signaling pathways [36]. Other studies have also shown the cytotoxicity of phytol on cancer cell lines as HL60 [37] which was due to induction of apoptosis [38].

Antibacterial activity of* L. pubescens* samples was evaluated by MIC determination for some bacteria commonly known to cause human infection. Results are reported in Table 3. MIC values ranged from 125 *μ*g/mL to 1000 *μ*g/mL. Ethyl acetate and hydromethanolic fractions showed the highest antibacterial activity against gram-negative bacteria* Enterobacter cloacae* (125 *μ*g/mL). Therefore, the overall antibacterial activity was not very significative. Among the seven bacterial strains tested, the gram-negative bacterium* E. cloacae* was the most sensitive to the extract and fractions, while* S. aureus* and* Enterococcus faecalis* were the most resistant. Previous works have demonstrated that sitosterol is inactive for several bacteria strains as some evaluated in this study [39, 40]. Phytol was shown to inhibit the growth of* S. aureus* [41]. However this activity was not observed in this work.

## 4. Conclusions

The results obtained represent a worthwhile contribution to biological and chemical knowledge of a traditional medicinal plant from Brazilian flora and indicated that* Lacistema pubescens* is a great candidate for further activity-guided fractionation in the search for new active therapeutic compounds.

## Figures and Tables

**Table 1:** Antileishmanial and antiproliferative activity of the methanolic extract and fractions of *L. pubescens*.

Samples	IC_50_ values (*µ*g/mL)^b^					
	Antileishmanial activity (promastigote forms)^a^				Antiproliferative activity	
	La	Lb	Lm	Lc	HL60	Jurkat
Methanolic extract	3.9 (±0.7)	45.6 (±2.1)	>100	>100	47.2 (±13.08)	>100
Hexane	3.5 (±0.9)	17.0 (±0.07)	4.2 (±0.8)	>100	39.7 (±1.37)	>100
Ethyl acetate	>100	>100	>100	>100	>100	>100
Hydromethanol	>100	>100	>100	>100	>100	>100
Amphotericin B^c^	0.4 (±0.05)	0.30 (±0.09)	0.3 (±0.09)	1.9 (±0.25)	—	—
Etoposide^d^	—^e^	—	—	—	0.02 (±0.01)	3.0 (±2.13)

Organism key:^ a^La: *Leishmania amazonensis*; Lb: *Leishmania braziliensis*; Lm: *Leishmania major*; Lc: *Leishmania chagasi*.

^b^IC_50_ values (concentrations inhibiting cell growth by 50%). Data are presented as median and 95% confidence interval (in parentheses).

^c,d^Control drug.

^e^ND: not done.

**Table 2:** Effect of the compounds on intracellular amastigotes of *L. amazonensis *and selectivity index.

Samples	Macrophages CC_50_ (*µ*g/mL) (95% C.I.)^a^	Amastigotes CC_50_ (*µ*g/mL) (95% C.I.)^a^	Selectivity index (SI)^b^
Methanolic extract	35.9 (±0.06)	8.1 (5.8–11.2)	4.4
Hexane	22.1 (±1.4)	6.9 (4.0–11.8)	3.2

^a^Data are IC_50_ values in *μ*g/mL and 95% confidence intervals are in brackets. ^b^Selectivity index (SI) was calculated by dividing the CC_50_ of macrophages by the IC_50_ values of amastigotes of *L. amazonensis*.

**Table 3:** Antimicrobial activity of the methanolic extract and fractions of *L. pubescens*.

MIC^a^(mg/mL)							
Samples	SA	PA	SD	ST	EC	EN	EF
Methanolic extract	1	0.5	0.5	0.5	1	0.5	1
Hexane	1	1	0.5	0.5	1	1	1
Ethyl acetate	1	0.5	0.5	0.5	0.5	0.125	1
Hydromethanol	1	0.5	0.250	0.250	0.5	0.125	1
Chloramphenicol^b^	0.02	>0.1	0.05	0.003	0.001	0.003	0.03

Microorganisms: SA: *Staphylococcus aureus*; PA: *Pseudomonas aeruginosa*; SD: *Shigella dysenteriae*; ST: *Salmonella typhimurium*; EC: *Escherichia coli*; EN: *Enterobacter cloacae*; EF: *Enterococcus faecalis*.

^a^MIC: minimum inhibitory concentration.

^b^Control drug.
